# Identification of the novel *COL5A1* c.3369_3431dup, p.(Glu1124_Gly1144dup) variant in a patient with incomplete classical Ehlers–Danlos syndrome: The importance of phenotype‐guided genetic testing

**DOI:** 10.1002/mgg3.1422

**Published:** 2020-07-28

**Authors:** Marco Ritelli, Valeria Cinquina, Marina Venturini, Marina Colombi

**Affiliations:** ^1^ Division of Biology and Genetics Department of Molecular and Translational Medicine University of Brescia Brescia Italy; ^2^ Division of Dermatology Department of Clinical and Experimental Sciences Spedali Civili University Hospital Brescia Italy

**Keywords:** atrophic scars, classical Ehlers–Danlos syndrome, *COL5A1*, next generation sequencing, Sanger sequencing, skin hyperextensibility

## Abstract

**Background:**

Classical Ehlers–Danlos syndrome (cEDS) is a connective tissue disorder mainly caused by heterozygous *COL5A1* or *COL5A2* variants encoding type V collagen and rarely by the p.(Arg312Cys) missense substitution in *COL1A1* encoding type I collagen. The current EDS nosology specifies that minimal suggestive criteria are marked skin hyperextensibility plus atrophic scarring together with either generalized joint hypermobility or at least three minor criteria comprising additional cutaneous and articular signs. To reach a final diagnosis, molecular testing is required. Herein, we report on a 3‐year‐old female who came to our attention with an inconclusive next generation sequencing (NGS) panel comprising all cEDS‐associated genes.

**Methods:**

Despite the patient did not formally fulfill the nosological criteria because the skin was only slightly hyperextensible, we made a cEDS diagnosis, mainly for the presence of typical atrophic scars. We investigated *COL5A1* intragenic deletions/duplications by Multiplex Ligation‐dependent Probe Amplification (MLPA), excluded the recessive classical‐like EDS type 2 by *AEBP1* Sanger analysis, and retested *COL5A1* with the Sanger method.

**Results:**

Molecular analyses revealed the novel *COL5A1* c.3369_3431dup p.(Glu1124_Gly1144dup) intermediate‐sized duplication with a predicted dominant negative effect that was missed both by NGS and MLPA.

**Conclusions:**

This report highlights that some cEDS patients might not display overt skin hyperextensibility and the importance of clinical expertise to make such a diagnosis in patients with an incomplete presentation. Our results also exemplify that NGS is not a fool‐proof technology and that Sanger sequencing achieves the diagnostic goal when there is a sufficiently clear phenotypic indication.

## Graphical abstract

We report on a pediatric patient with skin fragility suggestive for classical Ehlers–Danlos syndrome (cEDS) but without marked skin hyperextensibility, who came to our attention with unconvincing results of an external next generation sequencing (NGS) panel that included all cEDS‐associated genes. Based on a phenotype‐first approach, mainly due to the presence of typical atrophic scars, we made a clinical diagnosis of cEDS. We explored for an intragenic rearrangement in *COL5A1* by MLPA, ruled out the classical‐like EDS type 2 by sequencing of *AEBP1*, and finally resequenced *COL5A1* with the Sanger method that documented a novel intragenic duplication. Our findings show that in some cEDS patients the skin might not be markedly hyperextensible and that the cutaneous hallmark of the disorder is characterized by the presence of multiple, widened atrophic scars. This report is also a good example on how the new sequencing technologies are not foolproof and suggest that clinicians should look beyond negative NGS testing when there is a sufficiently clear phenotypic indication.

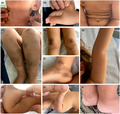

## 1 INTRODUCTION

Classical EDS (cEDS, MIM #130000, #130010), a heritable connective tissue disorder (HCTD) with an estimated prevalence of 1/20,000, is mainly characterized by cutaneous and articular involvement, but the clinical picture variably involves multiple organ systems (Bowen et al., 2017; Colombi, Dordoni, Venturini, Ciaccio, et al., 2017; Malfait et al., 2005, 2017; Ritelli et al., 2013; Symoens et al., 2012). Current major diagnostic criteria for cEDS are (1) skin hyperextensibility plus atrophic scars and (2) generalized joint hypermobility (gJHM) evaluated according to the 9‐point Beighton score (BS) (Beighton, De Paepe, Steinmann, Tsipouras, & Wenstrup, 1998). Minor criteria include easy bruising, soft, doughy skin, skin fragility, molluscoid pseudotumors, subcutaneous spheroids, hernia, epicanthal folds, gJHM complications, and an affected first‐degree relative. Minimal criteria for a cEDS diagnosis are major criterion 1 plus either major criterion 2 or 3 minor criteria (Bowen et al., 2017; Malfait et al., 2017).

Recognition of cEDS is generally not challenging, since most patients present with the typical cutaneous hallmarks. However, some do not and might remain undiagnosed or misdiagnosed by physicians with less experience in these disorders (Colombi, Dordoni, Cinquina, Venturini, & Ritelli, 2018). Differential diagnosis includes the molecularly unsolved hypermobile hEDS (MIM *130020) that shares with cEDS gJHM and more than a few cutaneous signs; however, hEDS patients usually show a lower grade of scarring and skin hyperextensibility and much more striking gJHM complications (Castori et al., 2015; Tinkle et al., 2017). In cases compatible with an autosomal recessive transmission, differential diagnosis comprises the classical‐like EDS (clEDS, MIM #606408) type 1, a.k.a. *TNXB* deficiency (MIM *600985) (Schalkwijk et al., 2001), and the recently defined clEDS type 2 (MIM #618000) caused by biallelic variants in *AEBP1* (MIM *602981) (Blackburn et al., 2018). The clEDS type 1 is generally distinguishable from cEDS for the absence of atrophic scarring (Brady et al., 2017; Rymen et al., 2019), whereas a more severe multisystemic presentation in clEDS type 2 should assist the differential diagnosis with cEDS (Ritelli et al., 2019; Syx et al., 2019). The dermatosparaxis (*ADAMTS2*, MIM *604539), cardiac valvular (*COL1A2*, MIM *120160), kyphoscoliotic (*PLOD*, MIM *153454; *FKBP14*, MIM *614505), and arthrochalasia (*COL1A1*, MIM *120150; *COL1A2*) EDS subtypes, also sharing with cEDS several cutaneous and articular issues, are generally distinguishable for the presence of specific hallmarks (Brady et al., 2017; Malfait et al., 2017).

In view of the significant overlap among the different EDS subtypes, a definite diagnosis of cEDS is established by the identification on molecular genetic testing of a heterozygous pathogenic variant in *COL5A1* (MIM *120215) and *COL5A2* (MIM *120190) encoding type V collagen or, less commonly, in *COL1A1*, encoding type I collagen (Brady et al., 2017; Malfait et al., 2017). The largest part of cEDS patients harbor *COL5A1* point mutations and the majority of these are null alleles leading to functional haploinsufficiency; a few intragenic rearrangements are also described (Colombi, Dordoni, Venturini, Ciaccio, et al., 2017; Ritelli et al., 2013). In *COL5A2*, structural variants exerting a dominant negative effect are the most common. In patients who fulfill the main clinical criteria of cEDS, the variant detection rate is about 90% (Ritelli et al., 2013; Symoens et al., 2012).

Until recently, genetic testing was mainly based on (serial) single‐gene testing by Sanger sequencing, that is, analysis of *COL5A1*, followed by *COL5A2*, gene‐targeted copy number variant (CNV) analysis, and search for the *COL1A1* c.934C>T p.(Arg312Cys) variant (Colombi, Dordoni, Venturini, Zanca, et al., 2017; Ritelli et al., 2013). Given the rapid improvements in next generation sequencing (NGS) technologies, custom laboratory‐designed multigene panels including at least all EDS‐associated genes are now used in almost all laboratories. Of note, as specific types of mutations may be lost due to technical limits, negative molecular testing does not exclude the diagnosis of cEDS.

In this work, we describe the clinical features and molecular diagnostic resolution by *COL5A1* Sanger sequencing of a girl presenting with an incomplete cEDS phenotype and a previously performed inconclusive NGS panel including all cEDS‐associated genes.

## 2 PATIENT AND METHODS

### 2.1 Ethical compliance

The reported data were obtained within a larger project on cEDS that was approved by the local Ethical Committee (Comitato Etico dell'ASST degli Spedali Civili, Brescia, Italy, registration number NP3873). The patient was evaluated at the specialized outpatient clinic for the diagnosis of EDS and related connective tissue disorders, that is, the Ehlers–Danlos Syndrome and Inherited Connective Tissue Disorders Clinic (CESED) at the University Hospital Spedali Civili of Brescia. Molecular analysis was achieved in the laboratory for genetic testing at the Department of Molecular and Translational Medicine of the University of Brescia. The patient's parents approved, by signed informed consent, molecular testing and publication of clinical data and photographs.

### 2.2 Molecular analyses

Molecular characterization was performed on genomic DNA purified from peripheral blood leukocytes by standard procedures. Deletion/duplication analysis of *COL5A1* was achieved through Multiplex Ligation‐dependent Probe Amplification (MLPA) with the SALSA MLPA P331 and P332 Probe‐Mixes, as previously described (Ritelli et al., 2013). All exons and intron‐flanking regions of *AEBP1* (NM_001129.4, NP_001120.3) and *COL5A1* (NM_000093.3, NP_000084.3) were amplified by PCR followed by bidirectional Sanger sequencing with the BigDye Terminator v1.1 Cycle Sequencing kit on an ABI3130XL Genetic Analyzer (Life Technologies, Carlsbad, CA, USA), as previously reported (Ritelli et al., 2013, 2019). The sequences were analyzed with the Sequencher 5.1 software (www.genecodes.com) and variants were annotated according to the Human Genome Variation Society nomenclature by using the Alamut Visual software version 2.15 (www.interactive‐biosoftware.com).

## 3 RESULTS

### 3.1 Clinical report

The family came to our attention for posttest genetic counseling, that is, interpretation of the results of an NGS panel performed by an external laboratory and clinical reevaluation of the proband with a HCTD suspicion. The affected individual was a 3‐year‐old girl, born to healthy unrelated parents from Italian origin and had a healthy sister. She was born at term after an uncomplicated pregnancy and delivery. Birth parameters were within the normal range (length 46 cm, weight 2315 g, and head circumference 34 cm). Postnatal neuro‐psychomotor development was normal. Clinical history was remarkable for JHM, a surgically treated umbilical hernia, mild astigmatism, and propensity to develop ecchymoses either spontaneously or upon minimal trauma. Considering this latter sign and normal blood coagulation parameters, a pediatric dermatologist, at 2.5 years of age, suspected a vascular HCTD and requested an NGS panel (*ACTA2*, MIM *102620;* CHST14*, MIM *608429; *COL1A1*;* COL1A2*;* COL3A1*, MIM *120180;* COL5A1*;* COL5A2*;* ELN*, MIM *130160;* FBN1*, MIM *134797;* FBN2*, MIM *612570;* FLNA*, MIM *300017;* MYH11*, MIM* 160745;* MYLK*, MIM *600922;* NOTCH1*, MIM *190198;* PLOD1*;* SLC2A10*, MIM *606145;* SMAD3*, MIM *603109;* TGFB2*, MIM *190220;* TGFBR1*, MIM *190181;* TGFBR2*, MIM *190182), which revealed two heterozygous variants both inherited from the father. Specifically, in *COL1A2*, associated with osteogenesis imperfecta (MIM #166210), cardiac valvular EDS (MIM #225320) arthrochalasia EDS (MIM #617821), and the recently defined COL1‐related overlap disorder (Brady et al., 2017; Marini et al., 2007; Morlino et al., 2020), the c.1295G>A, p.(Arg432Gln) missense variant (rs139446305, GnomAD 18/264778 individuals, no homozygotes, total MAF: C =0.00006798) was identified. In *FBN2*, associated with congenital contractural arachnodactyly (CCA, MIM #121050) (Meerschaut et al., 2020), the c.3565C>T, p.(His1189Tyr) variant (rs779690646, GnomAD 7/282780 individuals, no homozygotes, total MAF: C=0.000024758) was disclosed. According to the guidelines of the American College of Medical Genetics and Genomics (ACMG) (Richards et al., 2015) and by using the InterVar (Clinical Interpretation of Genetic Variants) tool (Li & Wang, 2017), both variants were classified as uncertain significance (ACMG class 3).

**Figure 1:**
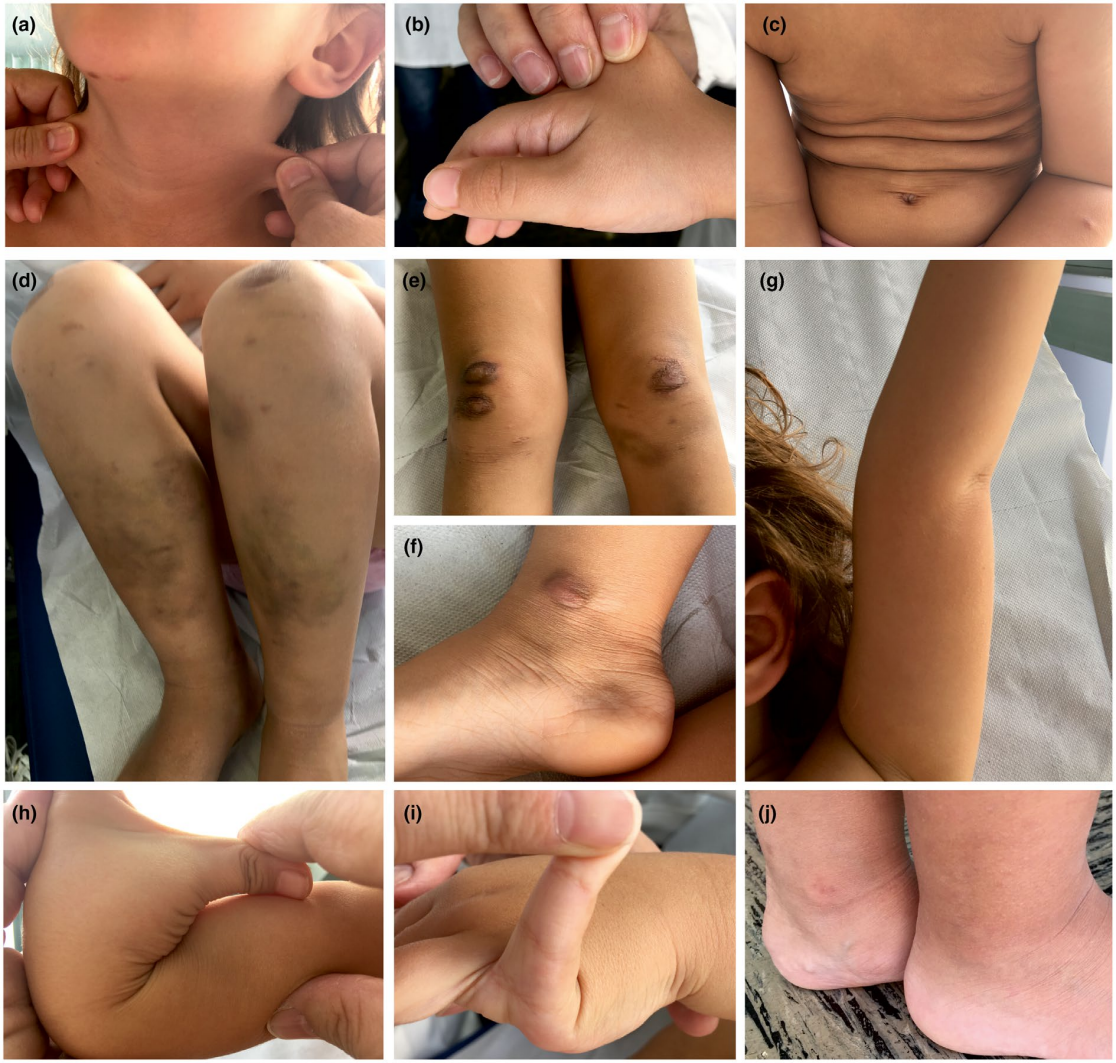
Clinical findings of the patient. Skin hyperextensibility at the neck and the dorsum of the hand (a, b); redundant, inelastic skin on the chest (c); easy bruising, valgus knees, and extensive scarring on knees, pretibial area, and ankle (d–f); hypermobility of the elbow (g), the thumb (h), and the fifth metacarpophalangeal joint (i); piezogenic papules of the heels (j)

At evaluation, the proband presented with soft, doughy skin that was slightly hyperextensible only at the neck and the dorsum of the hand (Figure 1a,b). Redundant, inelastic skin resembling cutis laxa on the lower chest was noted (Figure 1c). Widened atrophic, papyraceous, and hemosiderotic scars were present on knees, pretibial area, and ankles together with extensive easy bruising (Figure 1d–f). Small atrophic scars were present on the forehead and the chin. Passive hyperextension of the elbows to more than 10 degrees (Figure 1g), opposition of the thumbs to the volar aspect of the ipsilateral forearm (Figure 1h), dorsiflexion of the fifth metacarpophalangeal joints beyond 90 degrees (Figure 1i), and forward flexion of the trunk were observed (BS 7/9). Major complications of joint instability such as sprains, luxations/subluxations, and pain were absent, except for bilateral flatfoot. Other signs referable to the EDS spectrum were valgus knees (Figure 1e) and piezogenic papules of the heels (Figure 1l).

Overall, the patient did not fulfill neither the minimal suggestive criteria for any type of EDS according to the 2017 nosology (Malfait et al., 2017) nor the recently defined clinical score for a suspicion of CCA (Meerschaut et al., 2020). However, considering the presence of widened atrophic scars and gJHM, cEDS was the most probable clinical diagnosis, although the formal absence of the major criterion 1 due to the lack of significant skin hyperextensibility. Indeed, apart from the dorsum of the hand, which was stretchable over 1.5 cm, at the other four areas defined in the cEDS nosology, that is, neck, forearm, elbow, and knees, the degree of skin hyperextensibility did not reach the standardized cutoff values. In addition, the patient's skin was not markedly hyperextensible also at eyelid, palm of the hand, submandibular region, chest, and abdomen, which are other sites routinely assessed in our clinical practice (Colombi, Dordoni, Venturini, Ciaccio, et al., 2017). Minor criteria supporting the diagnosis of cEDS included easy bruising, skin texture, history of hernia, and bilateral flatfoot.

### 3.2 Molecular findings

**Figure 2:**
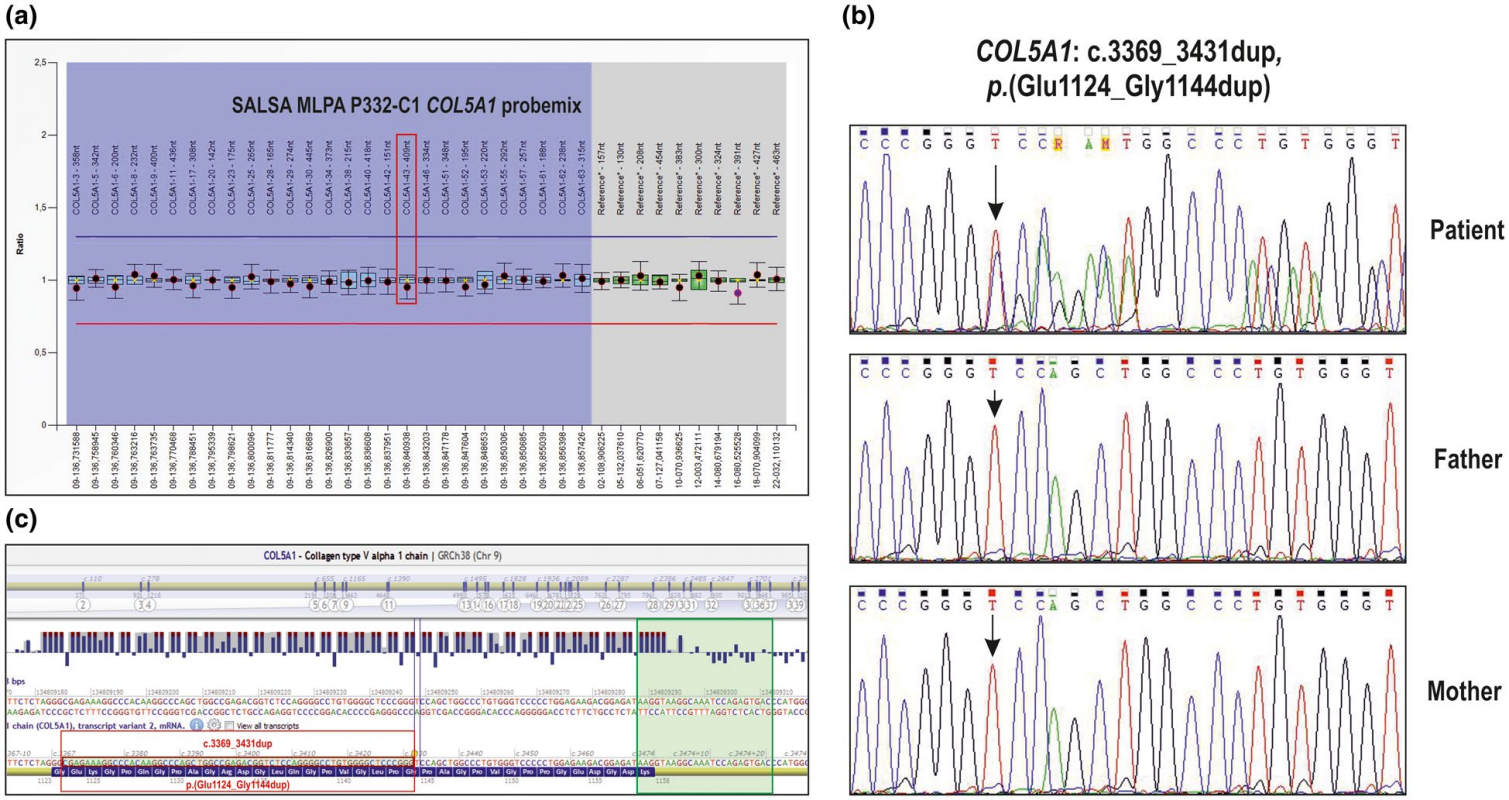
Molecular findings. (a) Multiplex Ligation‐dependent Probe Amplification (MLPA) results obtained by using the SALSA MLPA kits P332‐C1 showing normal copy number state of exon 43 (red box) of *COL5A1*. (b) Sequence chromatograms showing the position of the de novo c.3369_3431dup, p.(Glu1124_Gly1144dup) variant (arrow) identified in heterozygosity in exon 43 of *COL5A1* (seq. ref: NM_000093.3, NP_000084.3). (c) View of exon 43 of *COL5A1* with the Alamut Visual software version 2.15. The red box shows the 63 duplicated nucleotides (c.3369_3431dup) and the green box the partial sequence (24 nucleotides adjacent to the ligation site) of the MLPA probe covering exon 43, as indicated by the product description of the manufacturer

Considering that (i) the patient's clinical presentation was highly suggestive for cEDS, (ii) the rare variants identified by NGS were most likely not pathogenic, and (iii) CNV analysis of the NGS panel was not performed, we first implemented genetic testing with MLPA analysis of *COL5A1*, which did not identify any intragenic rearrangement (Figure 2a). As a second step, given that the family history was compatible with an autosomal recessive transmission, we performed *AEBP1* sequencing without disclosing any pathogenic variant. Finally, since quality and coverage results of the cEDS associated genes of the NGS panel were not available, we resequenced all exons and exon/intron boundaries of *COL5A1* with the Sanger method, according to our internal diagnostic flowchart (Ritelli et al., 2013). This analysis disclosed in exon 43 a de novo heterozygous duplication of 63 nucleotides c.3369_3431dup (Figure 2b) predicted to cause the in‐frame duplication of 21 amino acid residues [p.(Glu1124_Gly1144dup)] within the triple helical domain of the protein, thus, likely exerting a dominant negative effect. On InterVar, this variant has a default interpretation of “likely pathogenetic” that turns into “pathogenetic” (ACMG class 5) after specifying the de novo event. Since the variant was not reported neither in literature nor in any variant database, it was submitted to the EDS Leiden Open Variation Database (LOVD) (Dalgleish, 1998) (DB‐ID COL5A1_00218). Given the nature of the identified mutation, that is, a relatively large exonic duplication, we questioned why MLPA analysis was not able to detect this variant. To this end, we examined the SALSA MLPA probe sequences corresponding to exon 43, according to the manufacturer's product description. As shown in Figure 2c, the region covered by the relative probe is external to the duplicated nucleotides, thus, explaining the negative MLPA result.

## 4 DISCUSSION

In this work, we reported a child with skin fragility suggestive for cEDS but without marked skin hyperextensibility, who came to our attention with unconvincing results of an external NGS panel. Specifically, all cEDS‐associated genes resulted negative and two VUS were reported, which have not been critically interpreted neither by the laboratory nor by the referring clinician. Based on a phenotype‐first approach, we excluded the pathogenicity of these variants by bioinformatic analysis, searched for an intragenic rearrangement in *COL5A1* by MLPA, and ruled out the clEDS type 2 by direct sequencing of *AEBP1*. Molecular diagnostic resolution was finally achieved by *COL5A1* Sanger sequencing that documented a novel intragenic duplication.

Until recently, Sanger sequencing has been the gold standard in clinical laboratories for single‐gene tests, but this method reaches the diagnostic goal mainly when there is a clear phenotypic indication. In rare genetic disorders with clinical and genetic heterogeneity such as EDS, clinical and molecular diagnosis might be challenging and NGS approaches can be of great assistance. However, a critical issue is to decide which kind of NGS testing strategy is best suited for each clinical case. Concerning multigene panels, clinicians need to determine which panel is most likely to identify the genetic cause in the patient at the most reasonable cost while limiting not only the identification of VUS, but also pathogenic variants in genes not explaining the phenotype. The pediatric dermatologist who ordered the specific NGS panel for our patient mainly suspected (or sought to exclude) a vascular HCTD and did not specifically suppose cEDS. In addition, he was not supported by the laboratory's report in the variants' interpretation. Consequently, and despite both VUS were inherited from the patient's healthy father, he referred the family to our specialized clinic. This aspect offers clues for reflecting on the relevance of genetic analysis in the NGS era and highlights the risky drifts of clinical accuracy due to a simplistic application of NGS technologies in clinical practice. Indeed, while in the past genetic data did not drive diagnosis, but had a primarily confirmatory role, nowadays physicians often begin with genetic tests and the major challenge is to convert genetic data into a primary diagnostic tool. This implies the need of an essential change of the figure of medical geneticists, who must complement their skills with expertise in the clinical interpretation of NGS data. Likewise, bioinformaticians are mandatory in clinical laboratories, where they must team up both with clinicians and laboratory staff to optimize NGS data analyses and interpretation. Clinical bioinformatic systems require determination of variant calling sensitivity, specificity, accuracy, and precision for all variants reported in the assay and these quality criteria must be specified in the report. This was not the case in our patient and we can only speculate that the lack of identification of the 63 nucleotides duplication in *COL5A1* was due to technical limits, such as insufficient exon coverage or more probably as a result of short DNA sequencing reads alignment that is recognized, based on the specific computational tool used, to lack accuracy in the detection of intermediate‐sized deletions and insertions/duplications (Li & Durbin, 2009; Shigemizu et al., 2018).

From a clinical viewpoint, our findings offer perspectives for a possible update of the nosological diagnostic criteria for a cEDS diagnosis, especially concerning cutaneous signs. Even if the cEDS‐specific triad, that is, widened, atrophic scars, marked skin hyperextensibility, and gJHM is highly predictive for molecular confirmation of the diagnosis, this combination is not represented in all cases, as shown in our patient. Hence, the suspect of cEDS is not always driven by the traditional criteria but is rather gestaltic and based on the overall clinical presentation. In the past, the assessment of the cutaneous involvement lacked standardized methods to measure either skin hyperextensibility or to qualitatively assess skin texture and scarring; thus, these cardinal cutaneous hallmarks to diagnose cEDS were (and partly still are) subjective and mostly left to the practitioner's experience (Remvig et al., 2010). The 2017 revision of the EDS nosology partly tried to overcome this issue by defining both the sites of skin hyperextensibility and the relative cutoff values, mainly based on a previous work of our group in which we systematically evaluated several mucocutaneous features in a cohort of 62 cEDS patients with a defined molecular defect (Colombi, Dordoni, Venturini, Ciaccio, et al., 2017). In this cohort, we identified marked skin hyperextensibility in about 84% of the patients and the most stretchable areas were elbows, neck, knees, and dorsum of the hand. The patient reported here, who presented only moderate skin hyperextensibility at the dorsum of the hand, substantiates the concept that some cEDS patients might not display overt skin hyperextensibility and the importance of clinical expertise to make such a diagnosis in patients with an incomplete presentation.

Regarding skin fragility, even if atrophic scars are also very variable in clinical appearance and affected sites and can be identified also in other EDS subtypes and HCTDs (Castori et al., 2015; Colombi, Dordoni, Chiarelli, & Ritelli, 2015; Malfait et al., 2017; Meester et al., 2017; Ritelli et al., 2017), this sign should be basically considered characteristic of cEDS. In our previously published cEDS cohort, 95% of patients showed atrophic scars and the most common affected sites were knees, face, pretibial area, and elbows. Scars were widened in most patients and the majority presented with more than one type of scar, among which hemosiderotic were the most common (Colombi, Dordoni, Venturini, Ciaccio, et al., 2017). The presence of multiple papyraceous and hemosiderotic scars on knees and pretibial area in the present patient perfectly fitted with these observations and was the main reason for our choice to make a clinical cEDS diagnosis. In addition, the presence of a BS of 7/9, although gJHM it is common to all EDS subtypes, together with few further minor signs supported our decision to not confidence the negative NGS results. Overall, our findings suggest that in clinical practice atrophic scars should be considered self‐reliantly from hyperextensibility as major criterion 1. This proposal is corroborated not only by the present patient, but also by our previous findings indicating that the combination skin hyperextensibility plus widened, atrophic scars was observed in about 82% of patients (Colombi, Dordoni, Venturini, Ciaccio, et al., 2017).

## 5 CONCLUSION

In conclusion, our findings expand both the knowledge on the clinical features of cEDS and the *COL5A1* allelic repertoire. We corroborate the notion that in some patients the skin might not be markedly hyperextensible and that the cutaneous hallmark of cEDS is characterized by the presence of multiple, widened atrophic scars, which should address proper genetic testing in the presence of either skin hyperextensibility or gJHM or three of the minor criteria defined in the current EDS nosology. This report is also a good example on how the new sequencing technologies are not foolproof and suggest that clinicians should look beyond negative NGS testing when there is a sufficiently clear phenotypic indication.

## CONFLICT OF INTERESTS

All authors declare that there is no conflict of interest concerning this work.

## AUTHOR CONTRIBUTIONS

Marina Colombi and Marco Ritelli conceived the study. Marina Venturini and Marina Colombi performed the clinical evaluation of the patient, genetic counseling, and follow‐up; Marco Ritelli and Valeria Cinquina carried out the molecular analyses; researched the literature and prepared the manuscript; Marina Colombi edited and coordinated the manuscript. All authors discussed, read, and approved the manuscript.
